# Negative Correlation between Serum S100B and Leptin Levels in Schizophrenic Patients During Treatment with Clozapine and Risperidone: Preliminary Evidence

**Published:** 2016

**Authors:** Narjes Hendouei, Seyed Hamzeh Hosseini, Amin Panahi, Zahra Khazaeipour, Fatemeh Barari, Adeleh Sahebnasagh, Shahram Ala

**Affiliations:** a *Assistant Professor, Department of Pharmacotherapy, Faculty of Pharmacy and Psychiatry and Behavioral Sciences Research Center, Addiction Institute, Mazandaran University of Medical Sciences, Sari, Iran. *; b *Professor of Psychiatry, Psychiatry and Behavioral Sciences Research Center, Addiction Institute and Department of Psychiatry, Mazandaran University of Medical Sciences, Sari, Iran.*; c *Dr Chamran Hospital, Tehran, Iran. *; d *Brain and Spinal Cord Injury Research Center, Neuroscience Institute, Tehran University of Medical Sciences, Tehran, Iran. *; e *Pharm.D,Psychiatry and Behavioral Sciences Research Center, Addiction Institute and Department of Psychiatry, Mazandaran University of Medical Sciences, Sari, Iran.*; f *Student Research Committee, Department of Pharmacotherapy, Faculty of Pharmacy, Mazandaran University of Medical Sciences, Sari, Iran. *; g *Department of Pharmacotherapy, Faculty of Pharmacy, Mazandaran University Medical Sciences, Sari, Mazandaran, Iran.*

**Keywords:** S100B, Leptin, schizophrenic patients, Atypical Antipsychotics

## Abstract

Recently, extensive efforts have been made to understand the rate of energy expenditure and the weight gain associated with atypical antipsychotic treatment, including identification of markers of obesity risk. In recent years, leptin, an adipocyte hormone, has gained significant interest in psychiatric disorders. S100B has been considered as a surrogate marker for astrocyte-specific damage in neurologic disorders. Also, S100B has been detected in adipose with concentration as high as nervous tissue as a second release source. In this study we evaluated the relationship between S100B and leptin in schizophrenic patients under treatment with clozapine and risperidone.This study included 19 patients meeting the DSM-IV-TR criteria for schizophrenia, having body mass index (BMI) of 16- 25 kg/m^2^ and suffering schizophrenia for more than 3 years and from this study. Twenty five healthy controls were group matched for age and gender whose BMI was 16-25 kg/m^2^. Serum S100B and leptin levels and positive and negative symptom scale (PANSS) were assessed at admission and after six weeks. During the study, S100B showed a strong and negative correlation with leptin (r = -0.5, P = 0.01). Also, there were negative correlation between serum S100B level and PANSS negative subscale after 6 weeks of treatment (r = -0.048, P = 0.8).

Positive correlation between leptin level and PANSS suggested a potential role for leptin which can mediate the link between antipsychotic induced weight gain and therapeutic response in schizophrenia.

## Introduction

Schizophrenia is a serious disorder with a worldwide prevalence of about one percent and is associated with a 20% higher mortality rate compared to the general population (1). Antipsychotic drugs are the first line of treatment for schizophrenia (2, 3). There are two classes of Antipsychotic (AP) medications in therapeutic protocols. Typical APs, like haloperidol, can induce extrapyramidal side effects with a much higher rate compared with Atypical APs like risperidoneand clozapine, have lower incidences of extrapyramidal side effects (4). Current management of schizophrenic patients involves increasing use of AAPs over the last decade (1). Treatment with these agents is accompanied with excessive weight gain, hyperlipidemia, and development of new-onset diabetes that reveal a higher risk for cardiovascular diseases which lead to an enhanced noncompliance, morbidity and mortality (5). Recently, extensive efforts have been made in understanding the rate of energy expenditure and weight gain associated with AAP treatment, including the identification of markers of obesity risk (4). Among these markers, leptin, an adipocyte hormone, has recently received significant interest in psychiatric disorders (4, 5). This hormone plays an important role in the regulation of food intake, energy homeostasis and body weight (6). Resistance to leptin could result in metabolic conditions and weight gain. Some current studies show that treatment with SGAs increase leptin resistance (7).

S100B, a calcium-binding protein, is mainly synthesized by and released from astrocytes and oligodendrocytes (8, 9). It helps protein phosphorylation, cytoskeleton assembly, ca^+2^ homeostasis, transcription factors and glucose metabolism (9). S100B has been considered as a surrogate marker for brain and astrocyte-specific damage or dysfunction in neurologic disorders such as stroke and traumatic brain injury (8-10). S100B has also been found in adipose with concentration as high as nervous tissue as second release source (9, 10) and is closely related to the regulation of cellular energy metabolism and lipolysis (5). A few recent studies found increase in S100B levels in schizophrenic patients under treatment with AAPs which was due to abnormality in regulation of cellular energy metabolism, increase in adipose tissue mass and insulin resistance (10-12). To our knowledge, the relationship between changes in serum leptin, as a well-known adipose related factors, and S100B levels, as a surrogate marker for regulation of cellular energy metabolism, and symptoms improvement of schizophrenia and side effects associated with atypical antipsychotic drugs, specifically metabolic syndrome, have not been studied .In this study we evaluated the relationship between S100B and leptin, a well-known adipose related factor, in schizophrenic patients receiving clozapine and risperidone.

## Experimental

*Study Population*

This study was conducted at a psychiatric inpatient unit of a university-affiliated hospital, (between September 2010 and November 2012) in Sari in north of Iran. It was approved by the ethic committee of Mazandaran University of Medical Sciences (MAZUMS) and written informed consent was obtained from all participants’ guardians.

*Patients*

During the period from September 2010 and November 2012, eighty five patients with schizophrenia were registered into the trial but forty met the inclusion criteria. Subjects were included in the study if meeting DSM-IV-TR criteria (13) for schizophrenia, having BMI of 16- 25 kg/m^2^ and suffering schizophrenia for more than 3 years. 

The schizophrenia patients were compared with 25 age- and gender-matched healthy volunteers Who had BMIs of 16 to 25 kg/m^2^ as control group from general population for establishment of normal serum levels of S100B and leptin.

Patients with other psychiatric disorders, a prior history of neurologic disorders, acute or chronic illnesses known to affect the immune, endocrine or metabolic system like pulmonary, infectious, and coronary heart diseases, neoplasm, manifested diabetes, hyperlipidemia, history of substance abuse or dependence, dementia, severe trauma, suicide attempts, a previous history of cholesterol lowering treatment and with alimentary restriction or evidence of clinical malnutrition were excluded from the study. In control group, the aforementioned disorders and psychiatric disorders were excluded after taking a detailed history. The subjects did not take any concomitant medication. 

Thirteen patients were on risperidone and six on clozapine, for at least 6 months (on average 345 ± 5 mg chlorpromazine equivalents/day) at the time of blood sampling. During the study period only co medication with anticholinergic drugs and benzodiazepines were allowed. Fourteen subjects suffered from undifferentiated schizophrenia, four from paranoid schizophrenia and one from residual schizophrenia. All patients had same food regime during the study. All schizophrenic patients were managed based on the guideline of the American Psychiatric Association (14). One psychiatrists, independently, diagnosed schizophrenia according to the DSM-IV-TR criteria (13) and ICD-10 (15). The psychopathological status of schizophrenic patients was assessed with the positive and negative symptom scale (PANSS) (16) at baseline and after 6 weeks.

*Sample collection*

*Biomarker measurement*

Blood was drawn after an overnight fast by venous puncture at 7 A.M on admission and after six weeks. Each sample was centrifuged (3500 × g) for 15 min then the serum was separated and stored at -80˚C for further analyses. A complete differential blood cell count, including total cholesterol, triglyceride, LDL-cholesterol, HDL- cholesterol levels, fast blood sugar, systolic and diastolic blood pressure, and BMI were measured on admission and also after six weeks for all patients.

Serum S100B was analyzed using commercially available ELISA kit (BioVender, Modrice, Czech Republic) according to the manufacturer instruction.

Leptin levels were determined by an enzyme linked immunosorbent assay kit (mediagnost, Germany) according to the manufacturer instruction.

All samples were assayed in duplicate. 

*Statistical analysis*

All data were assessed for normality by one sample Kolmogorov-Smirnov test. Qualitative variables were recorded in frequency and percentage and quantitative variables in Mean ± SD (Standard Deviation). To compare continuous variables in two independent groups, we used t-test or Mann-Whitney U Test. Chi-Square test was applied to compare categorical variables. Also, paired t-test was used to compare two related continuous variables. The correlation between quantitative variables was made by spearman test. All statistical analyses were conducted using SPSS version 18 (SPSS Inc., Chicago, IL, USA) and P value of less than 0.05 was considered significant.

## Results

**Figure 1:**
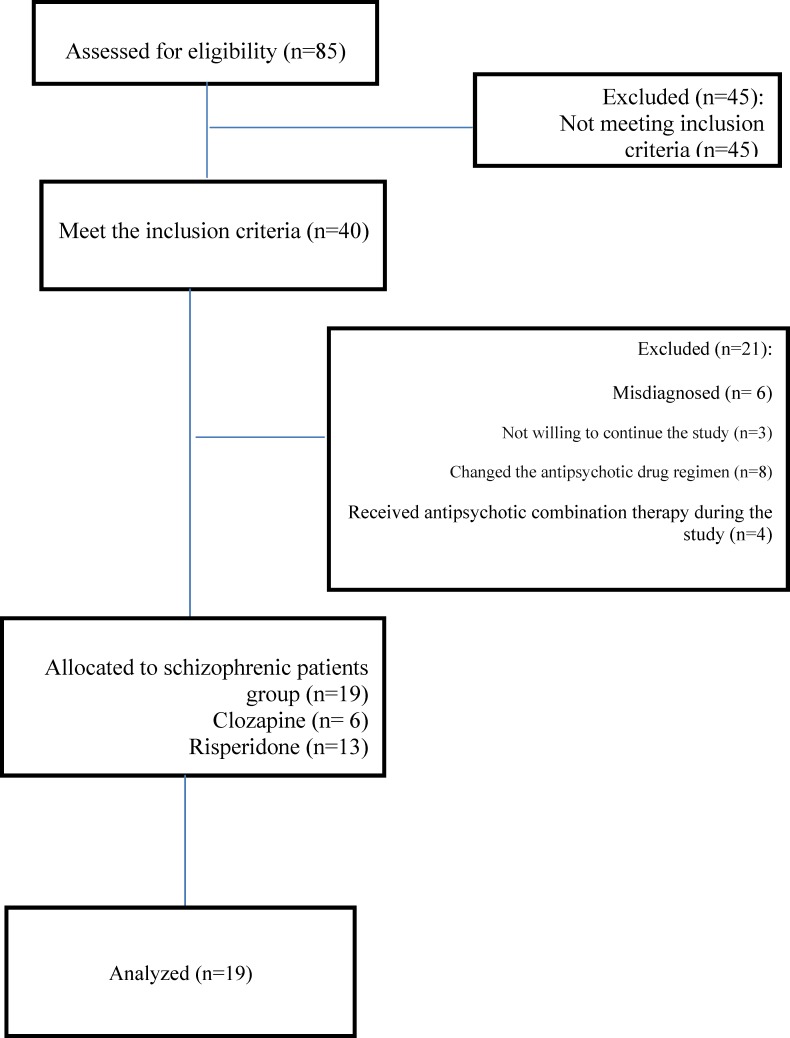
flow diagram of patients

During the period from September 2010 and November 2012, eighty five patients with schizophrenia were registered into the trial but forty met the inclusion criteria. Forty patients who met the inclusion criteria entered the study, of which three did not complete their study, six were misdiagnosed .Among all the patients who received antipsychotic drugs, eight patients, psychiatrists changed their antipsychotic drug regimen and four received antipsychotic combination therapy during the study .Finally nineteen patients completed the study (Figure 1). 

Baseline demographic and other baseline measurements between the study and control group are shown in Table 1.

**Table 1:** Baseline demographic and clinical measurements of schizophrenic patients and controls.

	**Schizophrenic patients(19)**	**Control (25)**	**P value**
Age(y)	34.05±9.9	34.2±8.3	0.9^a^
Male/Female (n)	13/6	18/7	0.7^b^
Duration of disease(y)	9.4±9.41	-	-
Subtypes of schizophrenia			
Undifferentiated type Residual type Paranoid type	14 1 4	-	-
BMI (kg/m2)	23.3±4.1	23.1±1.2	0.8^a^
Total Cholesterol (mg/dl)	173.3±38.7	227.7±43.2	<0.0001^a^
Triglyceride (mg/dl)	122.4±45.8	169.8±59.8	0.006^a^
LDL-C (mg/dl)	103.4±103.4	167.7±36.5	<0.0001^a^
HDL -C(mg/dl)	45.7±16.8	56.8±12.2	0.01^a^
FBS (mg/dl)	79.8±7.9	70.9±16.8	0.02^a^
S100B (pg/ml)	21.1±11.1	26.4±16.8	0.2^a^
Leptin (microg/ml)	9.01±11.74	11.7±17.2	0.3^c^
PANSS total score	85.3±24	-	-
PANSS positive (subscale score)	20.8±8.5	-	-
PANSS negative(subscale score)	25.3±7.8	-	-
PANSS general psychopathology (subscale score)	39±9.5	-	-

BMI: Body Mass Index, LDL-C: Low-density lipoprotein**-**Cholesterol, HDL: High-density lipoprotein-Cholesterol, FBS: Fasting Blood Sugar, PANSS: Positive and Negative Symptom Scale

* p < 0.05 considered significant

a : T Test,

b : Chi-Square Test ,

c : Mann-Whitney U Test

On admission, the lipid profiles in matched healthy controls were significantly higher compared to those of the schizophrenic patients (Table 1).

The mean serum concentration of S100B and leptin were 26. 4 ± 16.8 picog/mL and 11.7 ± 17.2 microg/mL, respectively for the control group. Healthy control group had higher initial serum levels of S100B and leptin but not significant at admission (P = 0.2, P = 0.3, respectively) (Table 1).

The lipid profiles and FBS levels increased during the study period, but only elevation of triglyceride level was significant (P = 0.002, Table 2).

**Table 2:** Clinical measurements at baseline and after 6 weeks in all schizophrenic patients

	**Baseline**	**After 6 wks**	**P value**
BMI (kg/m2)	23.3±4.1	23.7±4.3	0.1
Total Cholesterol (mg/dl)	173.3±38.7	189.7±37.7	0.2
Triglyceride (mg/dl)	122.4±45.8	186.6±137.5	0.03
LDL –C (mg/dl)	103.4±103.4	109.24±30.109	0.5
HDL –C (mg/dl)	45.7±16.8	45.45±12.7	0.9
FBS (mg/dl)	79.8±7.9	92.63±33.7	0.09
S100B (pg/mL)	21.1±11.1	17.1±5.6	0.2
Leptin (microg/mL)	9.01±11.74	8.3±8.4	0.8
PANSS total score	85.3±24	53.1±13.9	<0.0001*
PANSS positive (subscale score)	20.8±8.5	11.9±4.5	<0.0001*
PANSS negative(subscale score)	25.3±7.8	15.8±7.1	<0.0001*
PANSS general psychopathology(subscale score)	39±9.5	25.6±5.4	<0.0001*

* Paired t test

BMI: Body Mass Index, LDL-C: Low-density lipoprotein- Cholesterol, HDL:High-density lipoprotein-Cholesterol,FBS: Fasting Blood Sugar , PANSS:Positive and Negative Symptom Scale

-* p < 0.05 considered significant

Following intervention, the levels of S100B and leptin decreased in case group but this reduction was not significant (P = 0.2, P = 0.8, respectively) (Table 2). Our intervention significantly reduced PANSS total score and all subscale scores (positive, negative and general psychopathology) (P < 0.0001, Table 2).

The correlation between changes of serum S100B, leptin, BMI, lipid profiles, FBS and PANSS (total and sub scores) are illustrated in Table 3. During the study, S100B showed a strong and negative correlation with leptin (r = -0.5, P = 0.01, Table 3). This significant prediction of S100B by leptin could be highly attributed to its close dependence on adipose tissue mass like S100B. Also, there were negative correlation between serum S100B level and PANSS negative subscale after 6 weeks of treatment(r= -0.048, P = 0.8, Table 4).

**Table 4.** Correlation between serum S100B and Leptin level changes with PANSS subscale scores and PANSS total score during the treatment in all patients

	**PANSS positive subscale score**	**PANSS negative subscale score**	**PANSS general psychopathology subscale score**	**PANSS total score**
Serum S100B level changes	r=0.3 P=0.2	r=-0.048 P=0.8	r=0.05 P=0.8	r=0.06 P=0.7
Leptin (microg/ml)	r=-0.3 P=0.1	r=-0.023 P=0.9	r=-0.2 P=0.2	r=-0.3 P=0.1

-Spearman analysis

* -p < 0.05 considered significant

## Discussion

In this study we found a positive correlation between serum S100B level and PANSS but it was negative between serum S100B level and leptin with PANSS negative subscale. Also, our results showed significant negative correlation between changes in serum S100B and leptin levels. To our knowledge, this is the first study that was evaluated the relationship between S100B and leptin, a well-known adipose related factor, in schizophrenic patients treated with atypical antipsychotics.

S100B is a protein with 21 k Da molecular weight which is implicated in various intracellular and extracellular functional processes (18, 19). It is known that S100B plays important roles in cell proliferation and differentiation, cellular energy metabolism, and cytoskeletal modification. S100B may act as cytokine after secretion from glial cells, CD8+ lymphocytes and NK cells, activated monocytes and microglial cells (20, 21). Our finding is similar to studies of Yan Qi *et al* (22) and Rothermundt *et al* (23) who found no significant correlation between S100B and psychopathological symptoms. But in Rothermundt study (23), the majority of patients had received typical antipsychotics (70%) and 19.4% were medicated by atypical antipsychotics. Some studies have shown that persistently high S100B levels to be associated with negative symptoms, and patients with high serum S100B levels slowed psychopathological improvement upon treatment (24). Also, in our study, patients with higher S100B levels experienced more negative symptoms and slower response to treatment.

Leptin is a 16 KD peptide synthesized in white adipose tissue. Leptin can across blood brain barrier and is transported to the hypothalamus where it acts to limit food intake. Leptin can activate proopiomelanocortin (POMC) cells in arcuate nucleus and leads to increase in the release of melanocortin peptides. This peptide inhibits food intake and regulates metabolism by energy storage and insulin secretion (24). Leptin is considered to interact with some neurotransmitters such as histamine and serotonin that increase in serotonin receptor binding have been demonstrated to decrease food intake and an interaction between leptinergic and serotonergic systems in CNS (20, 25). Dopamine neurons have been shown to play a role in maintaining food intake. In humans, reduction in the concentration of dopamine metabolites in CSF is associated with an increase in leptin release which could reflect inhibition of dopamine release by leptin (24).

Some current studies show treatment with atypical antipsychotics increases resistance to leptin (7). Actually administration of atypical antipsychotics may result in increase of food intake, stimulating insulin release, post prandial hyperinsulinemia, and weight gain. Similarly to our study, Teff *et al* (20) showed the effect of weight gain due to atypical antipsychotics accompanied by elevated levels of triglyceride, LDL-cholesterol, HDL-cholesterol levels and fast blood sugar at onset of atypical antipsychotic treatment.

Recent investigations revealed an additional source for S100B. Steiner *et al* found the concentration of S100B in adipose tissue is as high as in nervous system and believed the elevated serum levels of S100B result from adipose tissue (17-19, 21).

S100B increases the intracellular energy reserved by activating glycolysis (fructose-1, 6 bisphosphonate aldolase) and glycogenolysis. Insulin has been shown to down regulate S100B expression in astrocyte cultures and rat brain with s100B binding to fructose-1, 6 bisphosphonate aldolase and phosphoglucomutase that may improve intracellular energy balance (18). In schizophrenic patients, disturbances in insulin signaling lead to the increased release of S100B and free fatty acids from adipose tissue (18, 26). Probably, an increased adipose tissue mass mainly attributed to the side effects of atypical antipsychotic medication, plays a major role in increased S100B levels in schizophrenic patients under atypical antipsychotic treatment too (17-19). However, it is worth pointing out that the source of circulating S100B remains unclear. 

Our results showed significant negative correlation between changes in serum S100B level and leptin that are in contrast with Steiner *et al* study who found a positive relationship between S100B and leptin levels. One possible reason could be that in their subjects S100B concentrations were particularly elevated at baseline and samples had BMI > 30. We found positive correlation between change in leptin level and PANSS which is in accordance to the studies of Venkatasubramanian G *et al* who suggested a potential role for leptin which can mediate the link between antipsychotic–induced weight gain and pleasure therapeutic response in schizophrenia (27).

The relationship between S100B, leptin and psychopathological symptoms in schizophrenic patients need further investigations. Despite the significant correlation between S100B and leptin, this initial finding should be reproduced in larger cohorts of unmedicated and medicated schizophrenic patients and different atypical antipsychotic medication subgroups (particularly effects of clozapine and olanzapine) and explore metabolic parameters (leptin and insulin levels) in both human serum and cerebrospinal fluid .

*Declaration of conflicting interests* *:* *None declared. Funding Acknowledgements:*

This research was financially supported by Mazandaran University of Medical Sciences grant.

## References

[1] Elias AN, Hofflich H (2008). Abnormalities in glucose metabolism in patients with schizophrenia treated with atypical antipsychotic medications. Am J Med.

[2] Scheen A, De Hert M (2007). Abnormal glucose metabolism in patients treated with antipsychotics. Diabetes Metab.

[3] Sentissi O, Epelbaum J, Olié J-P, Poirier M-F (2008). Leptin and ghrelin levels in patients with schizophrenia during different antipsychotics treatment: a review. A review Schizophr Bull.

[4] Jin H, Meyer JM, Mudaliar S, Jeste DV (2008). Impact of atypical antipsychotic therapy on leptin, ghrelin, and adiponectin. Schizophr Res.

[5] Ehrlich S, Salbach-Andrae H, Weiss D, Burghardt R, Goldhahn K, Craciun EM, Franke L, Uebelhack R, Klapp BF, Lehmkuh U (2008). S100B in underweight and weight-recovered patients with anorexia nervosa. Psychoneuroendocrinology.

[6] Atmaca M, Kuloglu M, Tezcan E, Ustundag B (2003). Serum leptin and cholesterol levels in schizophrenic patients with and without suicide attempts. Acta Psychiatr Scand.

[7] Haupt DW, Luber A, Maeda J, Melson AK, Schweiger JA, Newcomer JW (2005). Plasma leptin and adiposity during antipsychotic treatment of schizophrenia. Neuropsychopharmacology.

[8] Rothermundt M, Missler U, Arolt V, Peters M, Leadbeater J, Wiesmann M, Rudolf S, Wandinger KP, Kirchne H (2001). Increased S100B blood levels in unmedicated and treated schizophrenic patients are correlated with negative symptomatology. Mol Psychiatry.

[9] Gattaz WF, Lara DR, Elkis H, Portela LV, Gonçalves CA, Tort AB, Henna J, Souza DO (2000). Decreased S100-beta protein in schizophrenia: preliminary evidence. Schizophr Res.

[10] Schroeter ML, Abdul-Khaliq H, Frühauf S, Höhne R, Schick G, Diefenbacher A, Blasig IF (2003). Serum S100B is increased during early treatment with antipsychotics and in deficit schizophrenia. Schizophr Res.

[11] Gonçalves CA, Leite MC, Guerra MC (2010). Adipocytes as an important source of serum S100B and possible roles of this protein in adipose tissue. Cardiovascular Psychiatry and Neurology.

[12] Meshkani R, Adeli K (2009). Hepatic insulin resistance, metabolic syndrome and cardiovascular disease. Clin Biochem.

[13] American Psychiatric Association (2008). Diagnostic and Statistical Manual of Mental Disorders. DSM-IV-TR.

[14] American Psychiatric Association (1997). Practice guideline for the treatment of patients with schizophrenia. Am J Psychiatry.

[15] World Health Organization (WHO) (1993). Classification of Mental and Behavioural Disorders.Diagnostic Criteria for Research.

[16] KaySR, Flszbein A, Opfer LA (1987). The Positive and Negative Syndrome Scale (PANSS) for schizophrenia. Schizophr Bull.

[17] Steiner J, Myint AM, Schiltz K, Westphal S, Bernstein H-G, Walter M, Schroeter ML, Schwarz JK, Bogerts B (2010). S100B serum levels in schizophrenia are presumably related to visceral obesity and insulin resistance. Cardiovascular Psychiatry and Neurology.

[18] Steiner J, Bernstein H-G, Bogerts B, Gonçalves C-A (2013). Potential roles of S100B in schizophrenia. Rev Psiq Clin.

[19] Steiner J, Schroeter M, Schiltz K, Bernstein H, Müller U, Richter-Landsberg C, Muller WF, Walter M, Gos T, Bogerts B, Keilhoff G (2010). Haloperidol and clozapine decrease S100B release from glial cells. Neuroscience.

[20] Teff KL, Kim SF (2011). Atypical antipsychotics and the neural regulation of food intake and peripheral metabolism. Physiol Behav.

[21] Steiner J, Schiltz K, Walter M, Wunderlich MT, Keilhoff G, Brisch R, Bielau H, Bernstein H, Bogerts B, Schroeter ML, Westphal S (2010). S100B serum levels are closely correlated with body mass index: an important caveat in neuropsychiatric research. Psychoneuroendocrinology.

[22] Qi LY, Xiu MH, Chen DC, Wang F, Kosten TA, Kosten TR, Kosten TR, Zhang XY (2009). Increased serum S100B levels in chronic schizophrenic patients on long-term clozapine or typical antipsychotics. Neurosci Lett.

[23] Rothermundt M, Ponath G, Glaser T, Hetzel G, Arolt V (2004). S100B serum levels and long-term improvement of negative symptoms in patients with schizophrenia. Neuropsychopharmacology.

[24] Panariello F, Polsinelli G, Borlido C, Monda M, De Luca V (2012). The Role of leptin in antipsychotic-induced weight gain: genetic and non-genetic factors. J Obes.

[25] Raposo N, Ferreira A, Gattaz W (2011). Body mass index increase, serum leptin, adiponectin, neuropeptide Y and lipid levels during treatment with olanzapine and haloperidol. Pharmacopsychiatry.

[26] Barnwal A, Oza B, Patel V (2012). Metabolic side effects of antipsychotic agents: a prospective study in a teaching hospital. NHL JMS.

[27] Venkatasubramanian G, Chittiprol S, Neelakantachar N, Shetty TK, Gangadhar BN (2010). A longitudinal study on the impact of antipsychotic treatment on serum leptin in schizophrenia. Clin Neuropharmacol.

